# Two more Posterior Hox genes and Hox cluster dispersal in echinoderms

**DOI:** 10.1186/s12862-018-1307-x

**Published:** 2018-12-27

**Authors:** Réka Szabó, David E. K. Ferrier

**Affiliations:** The Scottish Oceans Institute, Gatty Marine Laboratory, School of Biology, University of St Andrews, East Sands, St Andrews, Fife, KY16 8LB UK

**Keywords:** *Hox11/13d*, *Hox11/13e*, Posterior Hox genes, Hox gene evolution

## Abstract

**Background:**

Hox genes are key elements in patterning animal development. They are renowned for their, often, clustered organisation in the genome, with supposed mechanistic links between the organisation of the genes and their expression. The widespread distribution and comparable functions of Hox genes across the animals has led to them being a major study system for comparing the molecular bases for construction and divergence of animal morphologies. Echinoderms (including sea urchins, sea stars, sea cucumbers, feather stars and brittle stars) possess one of the most unusual body plans in the animal kingdom with pronounced pentameral symmetry in the adults. Consequently, much interest has focused on their development, evolution and the role of the Hox genes in these processes. In this context, the organisation of echinoderm Hox gene clusters is distinctive. Within the classificatory system of Duboule, echinoderms constitute one of the clearest examples of Disorganized (D) clusters (i.e. intact clusters but with a gene order or orientation rearranged relative to the ancestral state).

**Results:**

Here we describe two Hox genes (*Hox11/13d* and *e*) that have been overlooked in most previous work and have not been considered in reconstructions of echinoderm Hox complements and cluster organisation. The two genes are related to Posterior Hox genes and are present in all classes of echinoderm. Importantly, they do not reside in the Hox cluster of any species for which genomic linkage data is available.

**Conclusion:**

Incorporating the two neglected Posterior Hox genes into assessments of echinoderm Hox gene complements and organisation shows that these animals in fact have Split (S) Hox clusters rather than simply Disorganized (D) clusters within the Duboule classification scheme. This then has implications for how these genes are likely regulated, with them no longer covered by any potential long-range Hox cluster-wide, or multigenic sub-cluster, regulatory mechanisms.

**Electronic supplementary material:**

The online version of this article (10.1186/s12862-018-1307-x) contains supplementary material, which is available to authorized users.

## Background

Hox genes encode a family of homeodomain-containing transcription factors that are renowned for conserved roles in the patterning of the anterior-posterior axis of bilaterian animals, and they may well have more ancient roles in animal axial development [1, 2]. Hox genes often occur in ordered clusters and exhibit spatial and/or temporal collinearity, wherein their order in the cluster matches their order of expression in the embryo [3, 4].

**Fig. 1:**
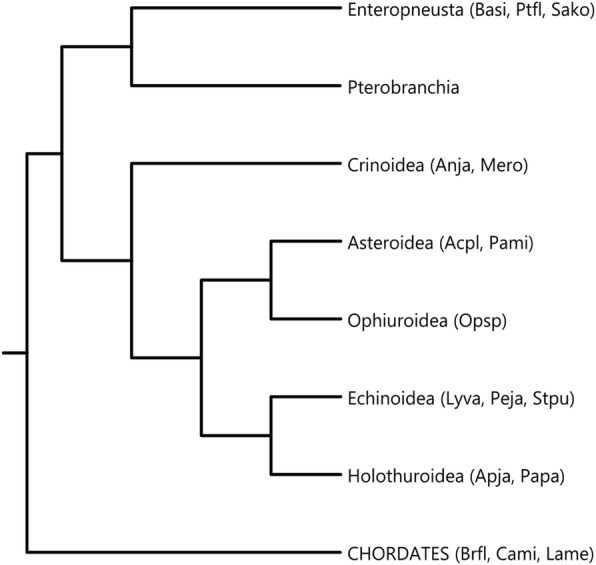
Schematic phylogenetic tree of Ambulacraria with chordates shown as the outgroup. Species used in this study are indicated in brackets next to their respective clades. Species abbreviations: Acpl, *Acanthaster planci*, Anja, *Anneissia japonica*, Apja, *Apostichopus japonicus*, Basi, *Balanoglossus simodensis*, Brfl, *Branchiostoma floridae*, Cami, *Callorhinchus milii*, Lame, *Latimeria menadoensis*, Lyva, *Lytechinus variegatus*, Mero, *Metacrinus rotundus*, Opsp, *Ophiothrix spiculata*, Pami, *Patiria miniata*, Papa, *Parastichopus parvimensis*, Peja, *Peronella japonica*, Ptfl, *Ptychodera flava*, Sako, *Saccoglossus kowalevskii*, Stpu, *Strongylocentrotus purpuratus*. Tree topology follows [61]

Echinoderms (which along with hemichordates constitute the Ambulacraria, see Fig. 1) occupy a key position in studies on the evolution of Hox gene organisation, not only because of the amenability of these organisms to molecular genetic research, but also because they provided the clearest example of intact but Disorganized (D) clusters, according to the classification system of Duboule [4]. That is, the echinoderm Hox genes exist in a cluster in the genome but the order and orientation of the genes within the cluster is rearranged relative to what is presumed to be the ancestral configuration. In recent years, the quantity and quality of available information on ambulacrarian Hox genes has increased dramatically. Beginning with the sequencing of the first sea urchin genome and the characterisation of its curiously scrambled Hox cluster [5], a series of studies brought an improved understanding of Hox gene complements and Hox cluster organisation in these animals [6–12]. The unconventional Hox cluster of sea urchins turned out to be a lineage-specific oddity, with both enteropneust hemichordates [9] and at least some non-echinoid echinoderms [10, 12] possessing intact, canonically ordered Hox clusters. However, even these more canonical forms of ambulacrarian Hox cluster still exhibited some levels of disorganisation due to gene loss and inversion of individual genes.

This wealth of new data has a potential to shed new light on the controversial evolutionary history of the Posterior Hox genes in deuterostomes. Posterior Hox genes, related to *Hox9* and above in vertebrates, have undergone a dramatic expansion in the deuterostome clade. Deuterostome species investigated so far have been found to possess at least four of these genes, in contrast to the one or two common among their protostome cousins. *Xenoturbella* and acoelomorphs, which possess small Hox complements with only a single Posterior in each species examined [13–17], have been classified as deuterostomes in some phylogenomic studies ([18, 19], but see [20]), but even if this placement is correct, the simple body plans and minimal Hox complements of these animals, confirmed by a recent in-depth analysis [17] are clearly atypical of Deuterostomia.

The cause(s) of deuterostome Posterior Hox expansion are not clear, although some have hypothesised a connection between the proliferation of Posterior Hox genes in chordates and the appearance of morphological novelties at the posterior end of the anterior-posterior (AP) axis, such as tails [21]. Equally unclear is the duplication history that led to the large Posterior Hox complements found in deuterostomes. Phylogenetic studies do not paint a clear picture, with generally poor resolution and variable topologies amongst the chordate *Hox9–15* genes and the ambulacrarian Posterior Hox complements of *Hox9/10* and *Hox11/13a-c* [5, 6, 8, 9, 22–27]. As early as 2000, this lack of resolution led Ferrier et al. [22] to suggest that the relatively high evolutionary rates of deuterostome Posterior Hox genes have largely obscured their true relationships, a hypothesis they dubbed Deuterostome Posterior Flexibility (DPF).

To begin to resolve such a thorny question, good taxon sampling and careful screening to identify the full Hox complements in each taxon are imperative. With the availability of expanding public genomic resources such as Echinobase [28], those requirements can be fulfilled with greater resolution and robustness than previously possible. Here, we report the presence of two previously overlooked Posterior Hox genes specific to echinoderms, which we name *Hox11/13d* and *e* based on their similarity to the well-known *Hox11/13b-c* genes of echinoderms and hemichordates. Both genes are shared by all extant echinoderm classes and absent from the currently published hemichordate genomes, and good evidence for developmental expression exists for one of them, which has previously been mis-classified. Intriguingly, neither gene is found within the Hox cluster in any of the echinoderm genomes in which assembly quality permits the investigation of linkage. In all species we studied, these novel genes reside on genomic scaffolds separate from those containing the canonical Hox cluster and are accompanied by at least one non-Hox gene. Motif and phylogenetic analyses confirm the status of *Hox11/13d* and *e* as distinct, echinoderm-specific Posterior Hox genes and the lack of close linkage with the Hox cluster requires that echinoderms be reclassified as having Split (S) clusters rather than simply Disorganized (D) clusters within the classification system of Duboule [4].

## Results

### Hox11/13d and Hox11/13e are novel Posterior Hox genes

*Hox11/13d* was first detected during an attempt to find orthologues of known echinoderm Hox genes in the ophiuroid *Ophiothrix spiculata*. After BLAST searches using homeodomain queries from *S. purpuratus* against the *O. spiculata* genome and the ophiuroid transcriptomes of Delroisse et al. [29] revealed the presence of three homeodomains highly similar to *Hox11/13b* and *c*, we conducted reciprocal BLAST searches against *S. purpuratus* as well as searches against the NCBI nr database. Thus, we found a previously undescribed gene model from *S. purpuratus* (NCBI accession XP_011680299.1) that could be aligned to one of the putative ophiuroid *Hox11/13b-c* type sequences over its full length. This sequence matched neither *Stpu-Hox11/13b* nor *c*, and mapped to scaffold 1168 in v4.2 of the *S. purpuratus* genome assembly, not scaffold 628, where the Hox cluster described by Cameron et al. [5] is located. Additionally, the novel gene showed high similarity to the sequence Tsuchimoto and Yamaguchi [30] identified as *Hox11/13c* in the sand dollar *Peronella japonica*.

Subsequent searches confirmed the existence of a *Hox11/13d* gene distinct from *Hox11/13b* and *c* in all other echinoderm genomes available at the time of study: the sea urchin *Lytechinus variegatus*, the sea cucumbers *Parastichopus parvimensis* and *Apostichopus japonicus*, the sea stars *Patiria miniata* and *Acanthaster planci*, and the crinoid *Anneissia japonica* (formerly *Oxycomanthus japonicus*).

**Fig. 2:**
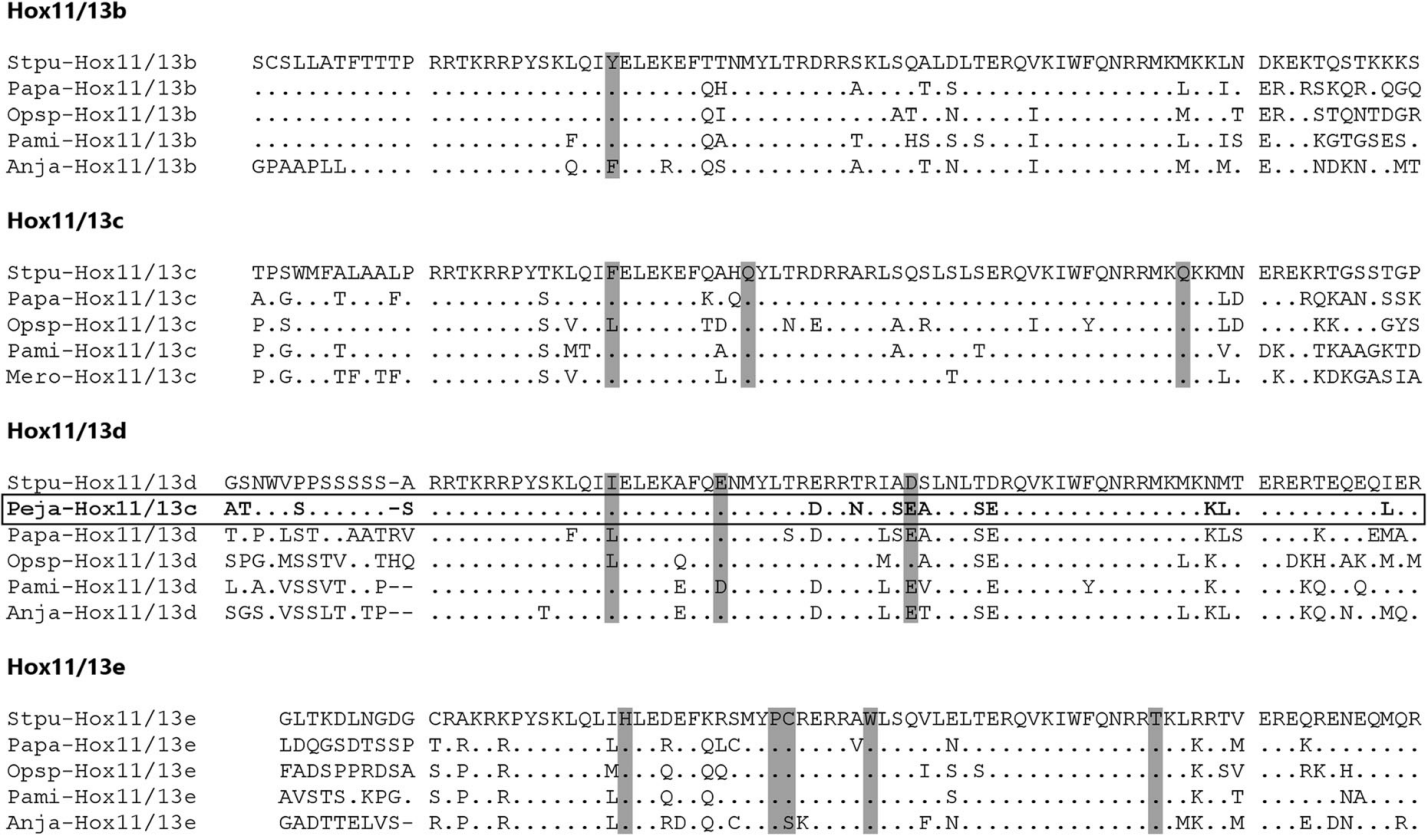
Alignment of echinoderm Hox11/13b+ homeodomains and flanking sequences. Identities to *S. purpuratus* are marked with dots. Potentially diagnostic residues within the homeodomain are highlighted in grey. Flanking sequences (N- and C-peptides) are separated from the homeodomain by a space. The misidentified “Hox11/13c” sequence from ref. [30] is boxed. Species abbreviations: Anja = *Anneissia japonica*, Mero = *Metacrinus rotundus*, Opsp = *Ophiothrix spiculata*, Pami = *Patiria miniata*, Papa = *Parastichopus parvimensis*, Peja = *Peronella japonica*, Stpu = *Strongylocentrotus purpuratus*

The gene we here name *Hox11/13e* was first identified by Thomas-Chollier et al. [27], who briefly mention a new “Hox11/13c-like” gene model (GLEAN_011798) that their survey detected in the *S. purpuratus* genome (in more recent versions of Echinobase, the gene model is called SPU_011798 and annotated as “Homeo2”). Although the phylogenetic analyses presented in the supplementary information for Thomas-Chollier et al. [27] consistently placed this model as a member of the *Hox11/13b-c* clade, their non-tree-based methods could not unequivocally identify the model as a Hox gene, and it is not discussed further in that study. BLAST searches against other echinoderm genomes clearly indicate the presence of the same very distinctive homeodomain in all species we studied (Fig. 2); *Hox11/13e* is therefore an echinoderm-wide gene.

We attempted to find orthologues for *Hox11/13d* and *e* in hemichordates; however, neither the genome of *Saccoglossus kowalevskii* nor that of *Ptychodera flava* yielded clear examples. In *P. flava*, no Posterior Hox genes were detected beyond the canonical four identified in previous studies [6, 8, 9]. In *S. kowalevskii*, there is a fifth, divergent Posterior Hox gene dubbed *AbdB-like* by Simakov et al. [31]. However, this sequence (accession: ALR88649.1) appears to be unique, and neither phylogenetic analyses nor sequence motifs clearly link it to either *Hox11/13d* or *e* (data not shown).

**Fig. 3:**
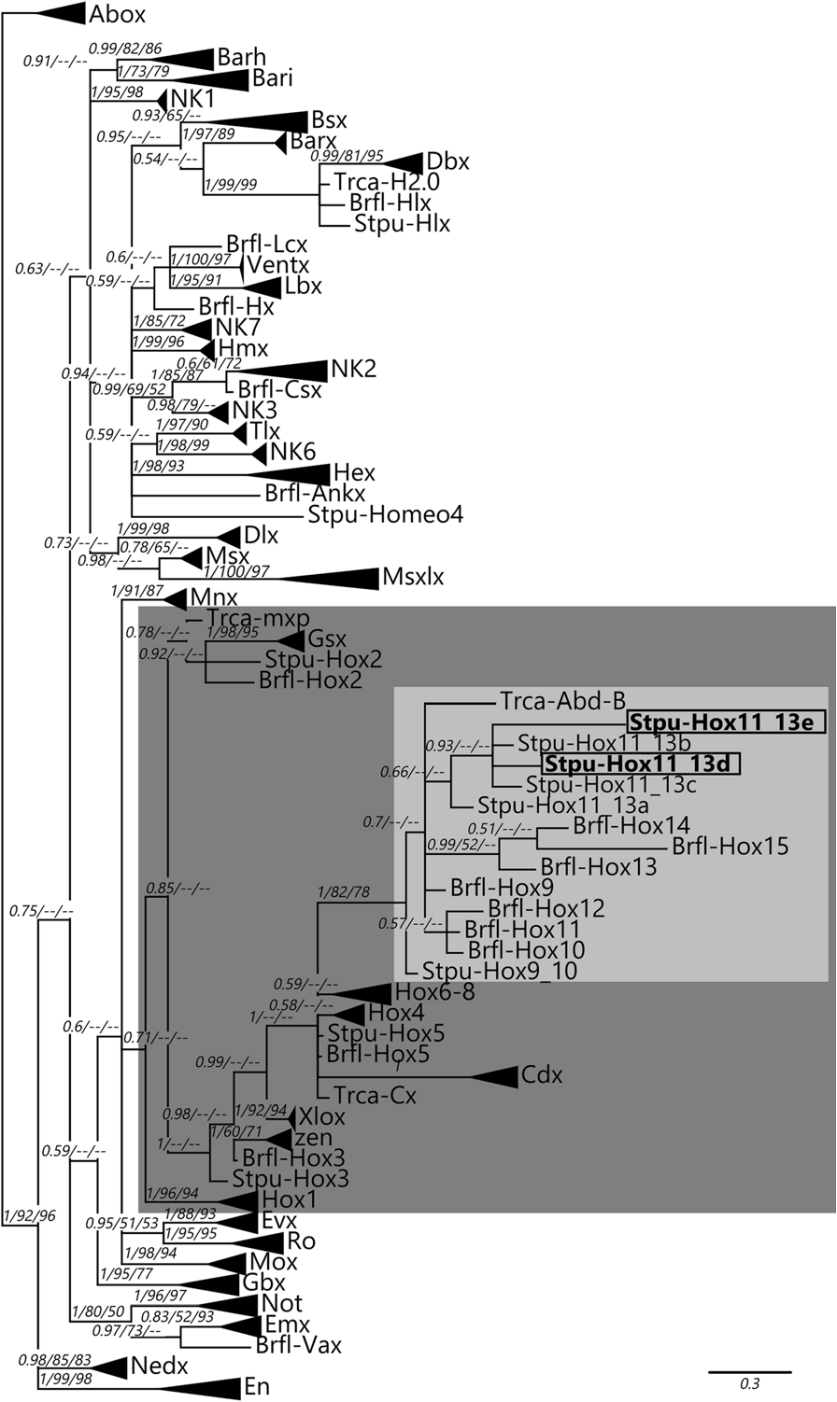
Bayesian tree of ANTP class homeodomains from amphioxus, beetle and sea urchin. The dark grey box indicates the Hox/ParaHox clade; Posterior Hox genes are highlighted in light grey and Hox11/13d and e are bolded and boxed. Support values above 50% from the Bayesian, maximum likelihood and NJ analyses are indicated next to the branches. Species abbreviations as before; Trca = *Tribolium castaneum*

Although the homeodomain of Hox11/13d is very similar to Hox11/13b and c, our putative Hox11/13e has a divergent homeodomain that does not show obvious affinity to previously recognised Hox genes (Fig. 2). We constructed phylogenetic trees of Antennapedia (ANTP)-class homeodomains from *Branchiostoma floridae*, *Tribolium castaneum* and *S. purpuratus* to confirm the assignment of the two novel sequences to the Hox clade. While resolution is generally poor within the Hox clade, all three tree reconstruction methods agree on the placement of Hox11/13d and e among the Posterior Hox genes (Fig. 3). Their position within that group is less certain. In our focused analyses of deuterostome Posteriors, their exact placement varies depending on the method used and whether flanking sequences are included in the alignment (Additional file 1 Fig. 4). Nevertheless, most analyses agree that echinoderm Hox11/13c, d and e are monophyletic (11/13b is always weakly supported and sometimes paraphyletic with respect to 11/13c). Also, most analyses recover an 11/13b+ clade, albeit not always with significant support (all trees can be found in Additional file 2), and all of them place the “Hox11/13c” sequence from *Peronella japonica* firmly within the Hox11/13d clade. The latter result provides a straightforward explanation for the “unstable” behaviour Tsuchimoto and Yamaguchi [30] observed from this gene in their own trees. It is notable that within the Hox11/13b+ group, the b and d clades generally receive weaker support than c and e and are sometimes recovered as paraphyletic, which may be due to the relatively conservative nature of the former pair (see also the section on motifs below).

**Fig. 4:**
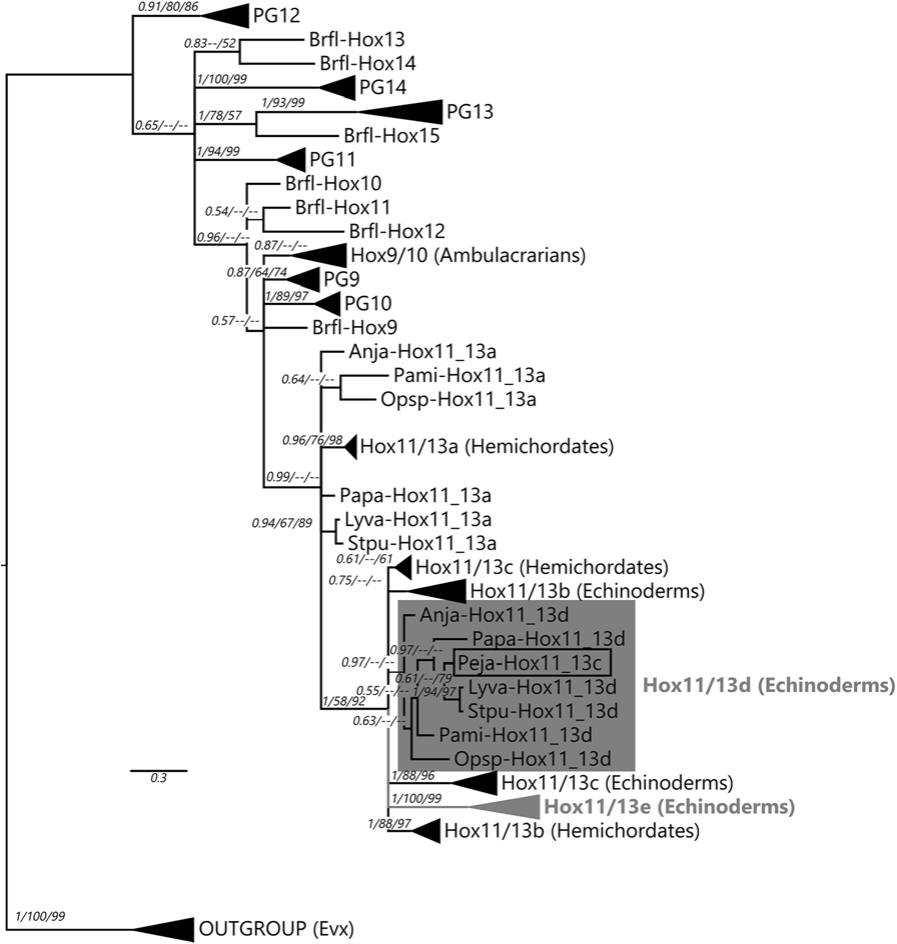
Bayesian tree of selected deuterostome Posterior Hox homeodomains and flanking sequences. Grey highlights indicate Hox11/13d and e, and the box shows the position of “Hox11/13c” from *P. japonica* within our Hox11/13d clade

### Hox11/13d and e are detached from the Hox cluster

**Fig. 5:**
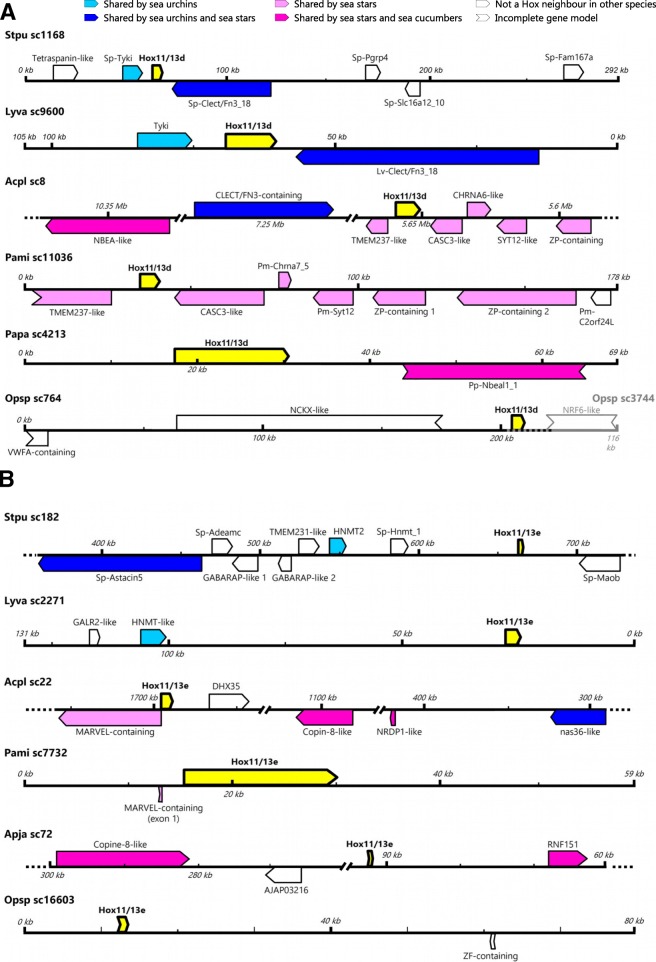
Non-Hox neighbours of *Hox11/13d-e*. **a**. Genomic scaffolds containing *Hox11/13d*. **b**. Genomic scaffolds containing *Hox11/13e*. Scale is indicated by the rulers on each scaffold. Scaffolds with reversed rulers have been flipped so that all Hox genes are shown in the same orientation. For sea cucumbers, the species with the most conserved neighbours is shown. The neighbourhood shown for *Opsp-Hox11/13d* is a composite of two overlapping scaffolds. Gene names prefixed with two-letter species abbreviations are taken directly from Echinobase annotations. Other gene names are based on Genbank annotations, BLAST hits and conserved domain content

One of the most striking observations about *Hox11/13d* and *e* in *S. purpuratus* is that neither is located on the scaffold containing the Hox cluster (scaffold 628 in the v4.2 assembly). Each of the novel Posterior genes is on a separate scaffold, linked to multiple non-Hox gene models (Table 1, Fig. 5). The same is the case for the high-quality v1.0 assembly of *Acanthaster planci*, in which the scaffold bearing *Hox11/13d* is almost 12 Mb long and contains over 400 annotated protein-coding genes (based on the automated NCBI genome annotation, release 100), with the Hox gene roughly in the middle of the scaffold (Table 1, Fig. 5a). *Acpl-Hox11/13e* is also situated in the middle of its 2.3 Mb scaffold, surrounded by 120 non-Hox genes. Other currently available echinoderm genomes have shorter scaffolds and less extensive annotations, but at least one non-Hox gene can be detected on each of the relevant scaffolds of the *L. variegatus, O. spiculata, P. miniata, A. japonicus* and *P. parvimensis* assemblies (Fig. 5). The genome of the crinoid *Anneissia japonica* (formerly *Oxycomanthus japonicus*) was only available in the form of raw reads at the time of this study. Thus, we focused on the other classes to check whether any of the non-Hox neighbours of the novel Posterior genes are conserved across taxa.

**Table 1:** Genome databases, assembly versions and Hox scaffolds used in this study

Species	Phylum/Class	Genome version	Database	Hox cluster scaffold(s)	Hox11/13d scaffold(s)	Hox11/13e scaffold(s)	Citations
*Anneissia japonica*	Echinodermata / Crinoidea	unassembled reads	NCBI SRA	N/A	N/A	N/A	None
***Lytechinus variegatus***	Echinodermata / Echinoidea	2.2	Echinobase	2296, 638, 378, 5296, 1336, 14,399, 33,366, 12,176, 234,558, 41,337, 1680	9600	2271	None
***Strongylocentrotus purpuratus***	Echinodermata / Echinoidea	4.2	Echinobase	628	1168	182	5
***Acanthaster planci***	Echinodermata / Asteroidea	1.0	NCBI genomes	15	8	22	10
***Patiria miniata***	Echinodermata / Asteroidea	2.0	Echinobase	2763, 4440, 5928, 12,174, 1848, 3114	11,036	7732	None
***Apostichopus japonicus***	Echinodermata / Holothuroidea	1.0 from [12]	Genedatabase.cn	420	872	72	11, 12
***Parastichopus parvimensis***	Echinodermata / Holothuroidea	1.0	Echinobase	391, 3188, 498, 1495, 4722, 3261, 5822, 317	4213	928	None
***Ophiothrix spiculata***	Echinodermata / Ophiuroidea	1.0 (contigs)	Echinobase	2313, 14,601, 543, 145, 887, 4941, 67,114, 5877, 10,227, 4461, 7983	764, 3374	21,026, 16,603	None
*Ptychodera flava*	Hemichordata / Enteropneusta	3.0	OIST	4932_cov136	N/A	N/A	31
*Saccoglossus kowalevskii*	Hemichordata / Enteropneusta	1.0	NCBI genomes	16,417, 1507, 26,913	N/A	N/A	31

We accessed databases through the following resources: NCBI genome portal and genomic BLAST [57], NCBI sequence read archive (SRA, [58]), Echinobase [28], the *Apostichopus japonicus* genome database [59] and the OIST Marine Genomics Portal [60]. Species used for neighbour analysis are indicated in bold italic

A handful of the non-Hox genes accompanying *Hox11/13d* and *e* were found to be shared between two or more examined species. Specifically, *Hox11/13d* is flanked by the same two genes in both sea urchins (Fig. 5a.). One of these neighbouring genes, encoding a large peptide containing multiple C-type lectin, fibronectin type 3 and CUB domains, is also on the *Hox11/13d* scaffold in *A. planci*, although there it is separated from the Hox gene by ~ 1.6 Mb and multiple other genes. The *Hox11/13d* scaffold of *P. miniata* has a gene content strongly conserved with the Hox neighbourhood of the corresponding *A. planci* scaffold (Fig. 5a).

Among the neighbours of *Hox11/13e*, *S. purpuratus* shares a putative orthologous histamine N-methyltransferase gene with *L. variegatus*. An additional *hnmt* gene is found in tandem with the first one in *S. purpuratus* but does not have a clear best match among the numerous similar sequences in the *L. variegatus* genome. A third *S. purpuratus* gene encoding an astacin-like metalloproteinase is conserved between the *Hox11/13e* scaffolds of that species and *A. planci*; considering that this gene is several hundred kilobases from *Hox11/13e* in *S. pupuratus*, the apparent lack of conservation with *L. variegatus* may be an artefact of the poorer quality of the latter’s genome assembly.

*P. parvimensis* and the two sea urchins do not share any non-Hox genes on their *Hox11/13d* and *e* scaffolds; however, the single non-Hox gene detected on each *P. parvimensis* scaffold is also present on the corresponding scaffold in *A. planci*. On the longer *Hox11/13e* scaffold of the *A. japonicus* assembly, a further gene encoding a copine-8-like sequence is a good match to a similar sequence in *A. planci*; however, despite its much larger size, the *Hox11/13d* scaffold of this species seems to lack the neurobeachin-like sequence shared between *P. parvimensis* and *A. planci.* None of the non-Hox genes detected next to *Hox11/13d* and *e* in *O. spiculata* are Hox neighbours in the other species.

### Diagnostic motifs

We ran the motif-finding program MEME on the non-homeodomain portions of representative deuterostome Posterior Hox sequences to see if we could discover diagnostic features for members of the Hox11/13b+ clade. We also hoped that such motifs could provide additional information to elucidate the tangled evolutionary history of this clade. Overall, the distribution of motifs in 11/13b + protein sequences is patchy (Additional file 3), with different motifs being shared by different groups of genes. Nonetheless, a few motifs may be useful in distinguishing members of this clade within echinoderms. One of these is the C-peptide, which is especially distinctive in Hox11/13e, where a long, strongly conserved region rich in charged residues follows the homeodomain. Hox11/13d has a similar, albeit more variable, charged C-peptide (Additional file 4 Fig. 2), which is largely absent from previously known members of the b + clade.

Another potentially informative motif is the hexapeptide and linker region, which is easily distinguishable between Hox11/13b, c and d, although any trace of a hexapeptide-like sequence (or indeed, any N-peptide conservation) appears to be missing from Hox11/13e (Fig. 2). Hox11/13b in non-crinoid echinoderms is characterised by a highly conserved N-peptide despite the loss of the tryptophan residue key to canonical hexapeptide function [30]. Likewise, this region is very similar in all echinoderm Hox11/13c sequences examined. The N-peptides of Hox11/13d sequences show less conservation, but they share a Ser/Thr-rich linker region. In hemichordates, the N-peptides of both Hox11/13b and c are most similar to that of echinoderm Hox11/13b except for the retention of the conserved tryptophan in the hemichordate sequences (Additional file 5).

**Fig. 6:**
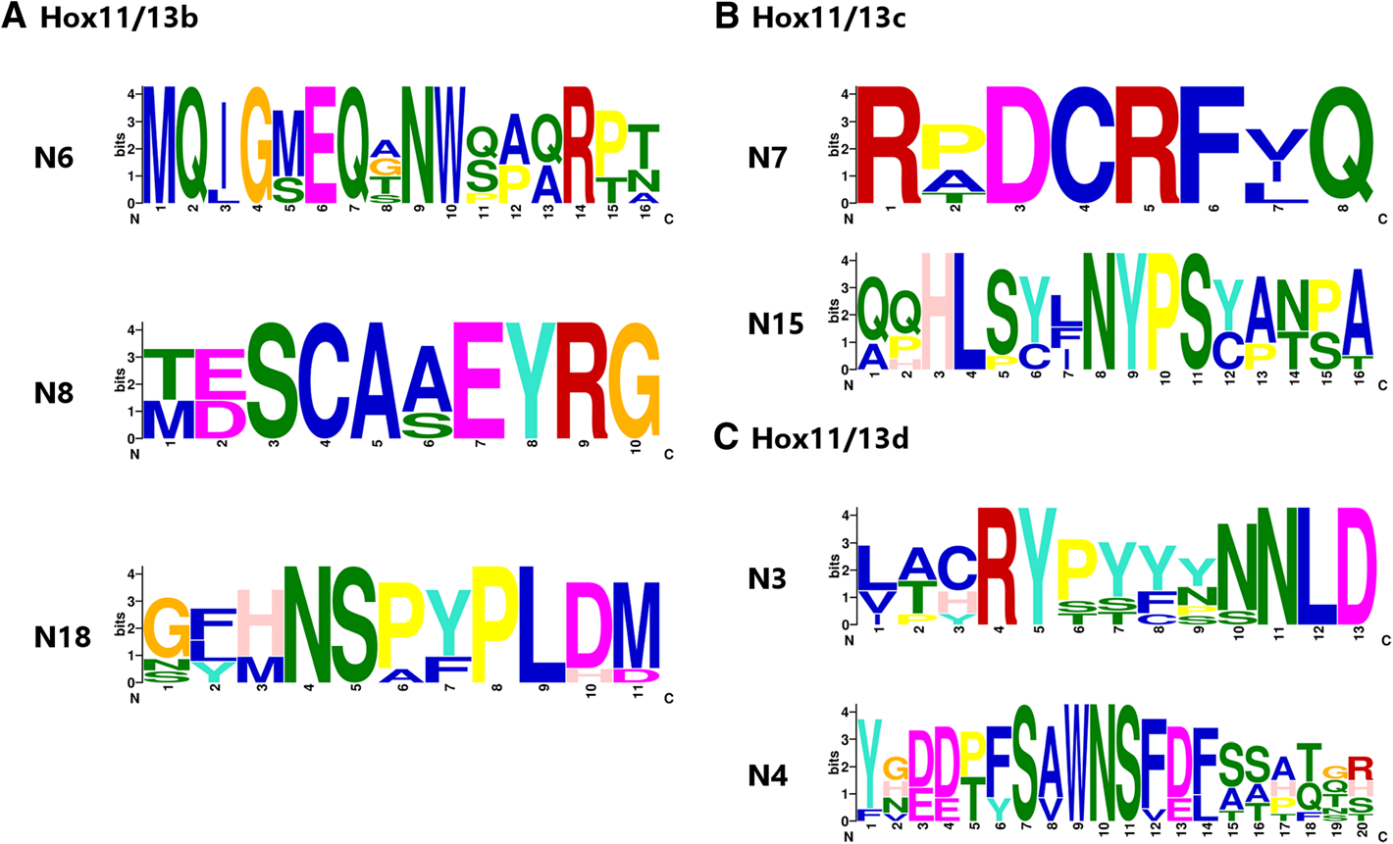
Potential diagnostic motifs from the non-homeodomain exons of echinoderm Hox11/13b-d. **a.** Motifs specific to Hox11/13b. **b**. Motif specific to Hox11/13c. **c.** Motifs specific to Hox11/13d. Logos were constructed from curated alignments of all echinoderm examples of each motif. For more information see Results and Additional files 3 and 4

Motifs from the non-homeodomain-containing first exons that may help distinguish Hox11/13b+ clade members in echinoderms are shown in Fig. 6 (see Additional file 4 for full alignments and placement in the peptide). Interestingly, the two exon 1 motifs “diagnostic” for Hox11/13d are also found in both hemichordate 11/13b+ members, while another motif (N18, see Additional file 4 Fig. 6) situated towards the end of exon 1 in echinoderm Hox11/13b is shared with hemichordate Hox11/13c but not b. Neither echinoderm Hox11/13c nor e share any motifs with hemichordates, and Hox11/13e appears to exhibit little to no sequence conservation outside of the homeodomain exon(s), although putative first exons of Hox11/13e are only known from sea urchins and sea stars at present.

### Evidence of expression

There is strong evidence for expression of *Hox11/13d* in multiple echinoderm classes. Most importantly, Tsuchimoto and Yamaguchi [30] provided good quality in situ data for this gene, although they worked under the assumption that it was *Hox11/13c*. Their experiments in *Peronella japonica* demonstrated expression in the vegetal plate during the blastula and early gastrula stages. There are no published *in situs* from other species, but transcripts of *Hox11/13d* are present in developmental transcriptomes of *S. purpuratus* ([32, 33], transcript ID WHL22.13708.0) and *L. variegatus* (transcript accession JI441003.1) available through Echinobase, the adult arm transcriptome of the ophiuroid *Ophiopsila aranea* [29], and the testicular transcriptome of *A. planci* ([34], SRX493873). We did not find *Hox11/13e* in any larval or adult transcriptomes except for a handful of reads in the *A. planci* testicular transcriptome.

## Discussion

In this study, we describe two novel echinoderm Hox genes with possible implications for Hox cluster evolution and the origin of the unusual body plan of Echinodermata. We named these genes *Hox11/13d* and *e* to indicate their relationship to the Hox11/13 genes previously described from both echinoderms and hemichordates. Our results increase the typical complement of echinoderm Posterior Hox genes to six, closer in number to the seven Posterior genes of the “archetypal” chordate amphioxus [26]. Additional, divergent sequences such as *AbdB-like* in *S. kowalevskii* and an as yet unnamed, unique Posterior Hox-like sequence we found in *O. spiculata* (data not shown) raise the possibility of even more unexplored, lineage-specific diversity in this iconic gene family.

The problem of Posterior Hox gene phylogeny in deuterostomes has endured ever since Ferrier et al. [22] first articulated it. Although that study largely focused on the problem as it pertains to chordates, the relationships of *Hox11/13b+* genes in ambulacrarians are equally difficult to resolve. Our work confirms that this problem cannot be easily solved with the addition of more taxa and sequences: despite including complete homeodomains and flanking regions of all types of 11/13 protein from all echinoderm classes and three hemichordates, our phylogenetic analyses still yield poorly resolved trees with conflicting topologies. Rather than illuminating its evolutionary history, the mosaic distribution of conserved sequence motifs outside the homeodomain (Additional files 3and 4 Fig. 6) indicates a high level of evolutionary flexibility in this clade.

Hox genes are best known for their conserved roles in patterning the bilaterian AP axis. In echinoderms, the ancestral AP axis is obscured by the pentameral symmetry of the adult body, but a spatially ordered “Hox vector” can still be discerned in the larval somatocoels of both echinoids [30, 35] and crinoids [7]. This vector incorporates *Hox7-Hox11/13b* in echinoids and *Hox5-Hox9/10* in crinoids, although Hara et al. [7] were unable to clone *Hox11/13b* from *M. rotundus*. Separate from this linear expression pattern, some Hox genes are also expressed in radial patterns in the adult rudiment; such radial expression has been reported for *Hox3* in *S. purpuratus* [36] and *Hox3, 5* and *11/13b* in *P. japonica* [30].

The conspicuous absence of Anterior and some Central Hox genes from the somatocoelar Hox vector, together with the rearrangement of the sea urchin Hox cluster with *Hox1–3* at the “posterior” end of the cluster [5], prompted Mooi and David [37] to hypothesise a link between cluster rearrangement and what they termed the axial, radially symmetrical region of a developing echinoderm adult. Building on Duboule’s [4] discussion of Hox cluster organisation and ordering as a possible evolutionary constraint, Mooi and David [37] and David and Mooi [38] suggested that the translocation of Anterior Hox genes in echinoderms permitted a delay in their expression and a dissociation from the AP axis, allowing their novel deployment as part of the developmental toolkit for radial adult structures. The above hypothesis predicted that the 5′ translocation of Anterior Hox genes would be ancestral to living echinoderms. However, the recent publication of Hox cluster data from sea stars [10] and sea cucumbers [12] suggests that it is, in fact, a peculiarity of echinoids and therefore not associated with the origin of pentameral symmetry [39].

All of the echinoderm Hox clusters described above fell into the “Disorganized (D)” category of Duboule’s [4] classification, meaning they were intact but relatively loosely organised, with losses, inversions and/or rearrangements within the cluster. Our findings reveal two novel genes that appear completely detached from the main Hox cluster even in the echinoderm species with the least disorganized cluster described to date [10]. Given that *Hox11/13d* and *e* both occur in every extant echinoderm class, the ancestral echinoderm Hox cluster may have been “Split (S)” sensu Duboule [4] instead of merely disorganized. Linkage data from crinoids, which form the sister group to all other living echinoderms, will be essential for testing this idea. A mostly intact Hox cluster which a subset of Posterior Hox genes have nonetheless escaped from is also seen in the annelid *Platynereis dumerilii* [40]; how closely this loss of cluster integrity parallels the situation in echinoderms remains to be seen.

*Hox11/13d* is expressed in embryonic stages of several echinoderms, likely an unusual trait for a Hox gene in this clade [30, 36]. Interestingly, the limited spatial expression data that exist for *Hox11/13d* [30] hint that despite its departure from the cluster, this gene may exhibit spatially coordinated expression with *Hox11/13b*, appearing in a domain more vegetal than the latter. Spatial collinearity of Hox gene expression is known to be at least partially independent of clustering. Residual spatial collinearity may persist even after complete Hox cluster disintegration [41, 42]. Conversely, Hox genes in canonically ordered clusters may evolve expression domains that break collinearity, as seen with *Hox6* and *Hox14* in the “archetypal” chordate amphioxus [43]. Thus, the regulatory, developmental and evolutionary significance of *Hox11/13d* and *e* being outside the Hox cluster is difficult to predict without more information on their expression and function.

Nothing is currently known about the expression and developmental roles (if any) of *Hox11/13e*. Unlike *Hox11/13d*, it is not present in any of the developmental transcriptomes we searched, suggesting that any developmental expression would happen at late stages that may be crucial for the development of the pentameral adult, or restricted domains that limit its detectability in transcriptome surveys. While Hox11/13d has a very similar homeodomain to Hox11/13b and c (Fig. 2) and shares several conserved motifs with the hemichordate members of the 11/13b+ group (Additional files 3 and 4), Hox11/13e has a highly distinctive homeodomain, so divergent that its original discoverers were not even certain it was a Hox gene [27]. In light of this, the high level of conservation seen in both its homeodomain and C-peptide across different echinoderm clades (Fig. 2) is intriguing, and so is the similarity of the C-peptide to Hox11/13d. As an echinoderm-specific Hox gene that is both highly unique and very conserved within echinoderms, *Hox11/13e* may prove especially interesting with regard to the evolution of the unusual body plan of this phylum.

Our discovery of two previously unrecognised (except for a brief mention of one of them in ref. [27]) Hox genes in the well-studied genome of the “model” echinoderm *S. purpuratus* highlights the continued need for in-depth studies focused on individual gene families in the age of big data. Such deep surveys may be particularly vital for Posterior Hox genes, whose higher levels of sequence divergence compared to most Anterior and Central Hox genes can make them difficult to catch in general homeodomain searches [22].

The improved taxon sampling resulting from the proliferation of “non-model” genome sequencing projects creates an unprecedented opportunity to chart the distribution of unusual members of key gene families such as *Hox11/13e*, an essential first step in understanding their role in body plan evolution. In combination with expression and functional studies, such surveys may shed new light on the origin of lineage-specific innovations.

## Conclusions

The two echinoderm Posterior Hox genes described here, *Hox11/13d* and *e,* have been largely neglected in previous work. These genes must have arisen early in echinoderm evolution since they are found in all extant echinoderm classes. Their genomic locations outside the Hox gene cluster, in all species for which data is available, requires the classification of the Hox clusters in these animals be revised. Echinoderms can no longer be considered as possessing Disorganized Hox clusters, but instead have undergone some dispersal of the Hox genes, thus making echinoderms another example of animals with Split Hox clusters. The impact of this Hox gene dispersal on the evolution of echinoderm body plans remains to be determined, but it is clear that *Hox11/13d* and *e* can no longer be controlled by any long-range gene regulatory mechanisms that may be operating within the remainder of the Hox cluster of sea urchins, sea stars, sea cucumbers, brittle stars and feather stars.

## Methods

### Hox gene surveys

During a general search for Hox gene sequences in the genome of the ophiuroid echinoderm *Ophiothrix spiculata* and transcriptomes of other ophiuroids [29], with sea urchin Hox cluster sequences as queries, an unexpected and thus far unannotated extra gene was found, here called *Hox11/13d*. Once it became clear that one of the transcriptomes and the *O. spiculata* genome both contained a previously undescribed Hox gene distinct from *Hox11/13b* and *c*, specific BLAST searches of all available echinoderm genomes were conducted using this novel sequence (chiefly its *S. purpuratus* orthologue, which has a probably complete RefSeq gene model under accession XP_011680299.1) as a query. *Hox11/13e* (then unnamed) was briefly mentioned in ref. [27] as a “*Hox11/13c-like*” gene in *Strongylocentrotus purpuratus*. We used the Echinobase gene ID given in that study (SPU_011798, currently annotated as “Homeo2”, an indeterminate ANTP-class gene) to search other echinoderm genomes for possible orthologues (see Additional file 6 for accessions, genomic locations and notes). To test whether *Hox11/13d* and *e* were echinoderm-specific or shared across the Ambulacraria, we also searched the genomes [31] of the hemichordates *Ptychodera flava* and *Saccoglossus kowalevskii* for additional Hox11/13b/c-like genes. Genome versions and databases used, along with the locations of the novel Hox genes, can be found in Table 1.

### Alignment and phylogenetic analysis

To ascertain that the novel genes we identified were indeed Posterior Hox genes, we created a reference alignment of ANTP-class homeodomains (Additional file 7) from the cephalochordate *Branchiostoma floridae*, the beetle *Tribolium castaneum* and the sea urchin *Strongylocentrotus purpuratus*. *B. floridae* and *T. castaneum* homeodomains were downloaded from HomeoDB [44] and checked by eye, while *S. purpuratus* homeodomains were extracted from the genome using the published homeobox survey of Howard-Ashby et al. [45] as a starting point and adding further sequences via BLAST searches of the v4.2 assembly. Homeodomains were manually aligned in Jalview 2.9 [46], and analysed with the neighbour-joining, maximum likelihood and Bayesian methods (see below for details).

In addition, more focused alignments of deuterostome Posterior Hox protein sequences were created to refine the placement of the novel genes within the Ambulacraria and Deuterostomia. We used Posterior Hox genes from selected echinoderm species with genomes available at the time of the analysis, as well as the “*Hox11/13c*” sequence from *Peronella japonica* published in [30], which we suspected might be a misidentified *Hox11/13d*. Hemichordates were represented by the acorn worms *Saccoglossus kowalevskii, Balanoglossus simodensis* [8] and *Ptychodera flava*, and chordates by the amphioxus *Branchiostoma floridae*, the coelacanth *Latimeria menadoensis* and the elephant shark *Callorhinchus milii*. In addition to homeodomains, approximately 12 amino acids of flanking sequence on either side of the homeodomain were also used in the full alignment. Where possible, N-terminal flanking sequences (herein, N-peptides) included the “vestigial” hexapeptide found in certain types of Posterior Hox proteins [47], with the conserved tryptophan as an anchor for alignment; attempts were also made to align C-peptides, although this could only be done within groups of orthologous proteins in most cases. Because we aligned flanking sequences to the extent this was possible, and because some Hox proteins (notably ambulacrarian Hox9/10) have C-peptides shorter than 12 residues, the exact lengths of the N- and C-peptides included vary. The full flanked alignment is available in Additional file 5. The flanked homeodomain alignment was created with MAFFT v7 [48] accessed through Jalview, and edited by eye.

Neighbour-joining trees were constructed in MEGA 7 [49], with pairwise deletion of missing data and 1000 bootstrap replicates. For maximum likelihood analyses, the PhyML 3.0 web service [50] was used with subtree pruning and regrafting as the search algorithm and 500 bootstrap replicates to assess tree robustness. Bayesian trees were generated with MrBayes v3.2.6 [51] using two parallel runs of four chains each with the default heating parameters. Bayesian analyses were run in increments of 1 million generations until the standard deviation of split frequencies between runs decreased below the recommended 0.01. This required 7 million generations for the ANTP and full Hox datasets, and 5 million for the homeodomain-only Hox alignment. The first 25% of each run was discarded as burn-in. ML and Bayesian analyses used the best model selected by modelgenerator v0.85 [52] for each dataset (JTT + I + Γ for the full Hox alignment and LG + Γ for the other two). In all cases, model selection was unanimous by all three information criteria employed by modelgenerator. NJ analyses were conducted with JTT + Γ, the highest-scoring model available in MEGA. Since MEGA does not estimate it automatically, the shape parameter α was manually set to the value given by modelgenerator (α = 0.36 for the ANTP dataset, 0.68 for the full Hox dataset and 0.39 for the homeodomain-only alignment).

### Motif analysis

MEME v4.12.0 [53, 54] was employed to search for potential diagnostic motifs outside the homeodomains of Posterior Hox proteins (Hox11/13b-c clade members in particular). In this analysis, each echinoderm class except crinoids was represented by a single species to avoid the problem of high overall similarity between closely related taxa obscuring potentially interesting motifs. The species chosen were *S. purpuratus* for Echinoidea, *Patiria miniata* for Asteroidea, *Parastichopus parvimensis* for Holothuroidea, and *O. spiculata* for Ophiuroidea. The crinoid data consisted of the complete protein sequences of *Metacrinus rotundus* Hox9/10 and Hox11/13c as determined by Hara et al. [7], supplemented by ORFs containing the homeodomains of Hox11/13a, b, d and e that were manually assembled from *Aneissia japonica* (formerly *Oxycomanthus japonicus*) raw genomic reads (NCBI Sequence Read Archive accession SRX447395). The non-homeodomain exons of these genes could not be obtained by BLAST searches of the read data. *S. kowalevskii*, a harrimaniid, and *B. simodensis,* a ptychoderid represented hemichordates, while the chordate data consisted of the Posterior sequences from *B. floridae* and a representative member of each paralogy group from *C. milii*, selected by visual inspections of PG alignments between *C. milii* and *L. menadoensis*. Homeodomains were excluded from the analysis, and the regions preceding and following the homeodomain were tested separately to avoid spurious motif detection across the site of the homeodomain. We searched for motifs between 6 and 20 amino acids long that occurred in at least two sequences with at most one occurrence per sequence, and used MEME’s default E-value threshold of 0.05 as a cutoff. The input sequences and Posterior Hox sequences from additional ambulacrarian taxa were then visually inspected for further instances of each motif, and MEME’s original alignments were manually curated before being converted into logos for publication. Weblogo v2.8.2 [55] with no small sample correction was used to generate the logos. Full motif alignments including partial instances omitted from the logos can be found in Additional file 4, and a summary of motif distributions in our dataset is given in Additional file 3.

### Non-Hox neighbours of Hox11/13d and e

In all examined genomes, we found that neither *Hox11/13d* nor *Hox11/13e* shared a scaffold with any other Hox gene. Therefore, we scanned the scaffolds containing *Hox11/13d* and *e* in *S. purpuratus*, *Lytechinus variegatus*, *Acanthaster planci*, *P. miniata* and *P. parvimensis* for non-Hox genes to further assess their separation from the main Hox cluster. The genome of *A. japonica* did not have long enough scaffolds to permit neighbour analysis and were therefore omitted. In the first instance, existing annotations from each genome database and (where available) transcripts mappable to the *Hox11/13d* or *e* scaffold were used to derive a preliminary list of neighbours for each Hox gene. These lists were curated to remove redundancy, extend gene models based on interspecific conservation and/or transcriptomic evidence where applicable, and check the locations of models on the scaffold. In all genomes except those of *Apostichopus japonicus* and *A. planci* (which has very large scaffolds and the best annotation quality of the species considered here), open reading frames longer than 100 amino acids were then scanned for further, unannotated genes using homology (species-specific Echinobase BLAST searches or general searches against the nr database) and the presence of conserved domains from the NCBI conserved domain database [56]. Finally, putative Hox neighbours from each echinoderm species were searched against the genomes of the other species to detect any conserved synteny. (For more information about Hox neighbours, see Additional file 6.)

## Additional files

Additional file 1: Bayesian tree of deuterostome Posterior Hox homeodomains without flanking sequences. Highlights and support values as in Fig. 4. (PNG 533 kb)

Additional file 2: Raw tree files (NEXUS or Newick) from our phylogenetic analyses. (ZIP 45 kb)

Additional file 3: Distributions of conserved motifs in ambulacrarian Posterior Hox proteins. (XLSX 15 kb)

Additional file 4: Motif alignments and locations. 1. Curated alignments of all echinoderm and hemichordate instances of the motifs detected by MEME in our deuterostome Posterior Hox datasets, 2. Example sequences showing typical motif locations within the protein. (PDF 109 kb)

Additional file 5: Full flanked Hox alignment used in phylogenetic analyses. The HD-only analyses (Additional files 1 and 2) used the same alignment without the flanking sequences. (FASTA 11 kb)

Additional file 6: Non-Hox neighbours of *Hox11/13d-e* in our study species. Sheets list names, gene/transcript IDs, genomic locations, conserved domain contents and best BLAST hits for the non-Hox protein-coding genes on each scaffold examined. Hox scaffold accessions and the exact locations of *Hox11/13d-e* within their scaffolds are also given, as well as the full inferred protein sequences of Hox11/13d-e. (XLSX 75 kb)

Additional file 7: ANTP class homeodomain alignment used in phylogenetic analyses. (FASTA 11 kb)
