# Supplementary figures and images for: Two more Posterior Hox genes and Hox cluster dispersal in echinoderms
Source: BMC Evol Biol. 2018 Dec 27;18:203. doi: 10.1186/s12862-018-1307-x (PMC6307216; doi:10.1186/s12862-018-1307-x)

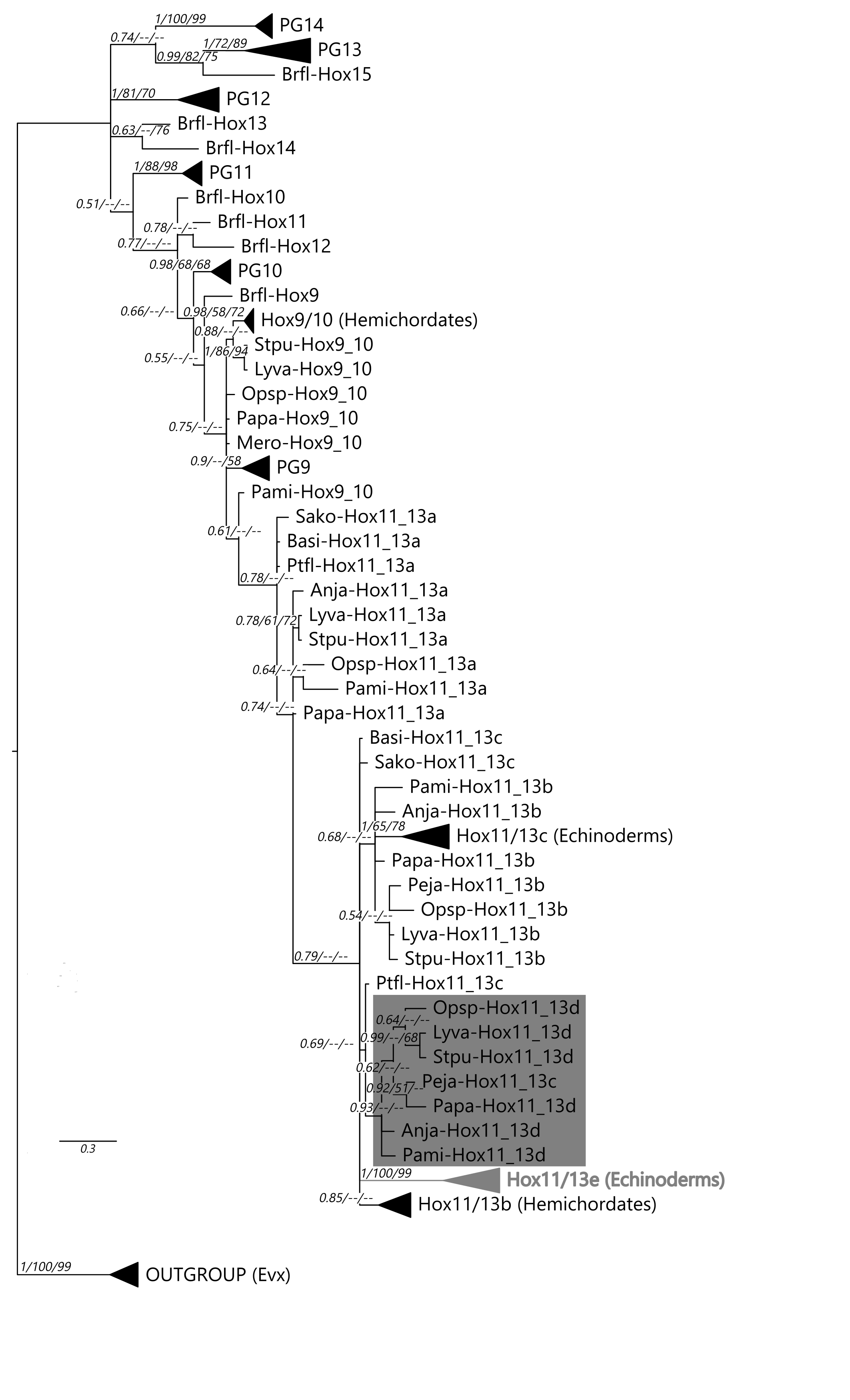

Supplement: Supplementary file 1 — Bayesian tree of deuterostome Posterior Hox homeodomains without flanking sequences. Highlights and support values as in Fig. 4. (PNG 533 kb) [file 12862_2018_1307_MOESM1_ESM.png]
