# Supplementary material for: Two more Posterior Hox genes and Hox cluster dispersal in echinoderms
Source: BMC Evol Biol. 2018 Dec 27;18:203. doi: 10.1186/s12862-018-1307-x (PMC6307216; doi:10.1186/s12862-018-1307-x)
Supplement: Supplementary file 4 — Motif alignments and locations. 1. Curated alignments of all echinoderm and hemichordate instances of the motifs detected by MEME in our deuterostome Posterior Hox datasets, 2. Example sequences showing typical motif locations within the protein. (PDF 109 kb) [file 12862_2018_1307_MOESM4_ESM.pdf]

Conserved motifs in ambulacrarian posterior Hox proteins.

1. Motif alignments. Motifs are listed and numbered in their order in the MEME outputs (“C” and “N” stand for C- and N-terminal datasets). Initial alignments generated by MEME were curated and expanded by eye. Motif instances detected by MEME are in bold; additional instances (complete or partial) found through visual inspection of the MEME dataset are in normal type, and further examples from species not included in the MEME dataset are in grey. For partial motifs, surrounding regions not conserved with the MEME motif are in lowercase. For species abbreviations see Fig. 1 in the main text.

C01

```
Stpu-Hox11/13e  EQRENEQMQRDMADLAMRFY
Lyva-Hox11/13e  EQRENEQMQRDMADLAMRFY
Pami-Hox11/13e  EQRENAQMQRDMANLAMRFY
Acpl-Hox11/13e  EQRENAQMQRDMANLAMRFY
Papa-Hox11/13e  EKRENEQMQRDMASLAMKFY
Apja-Hox11/13e  EKRENEQMQRDMASLAMKFY
Opsp-Hox11/13e  ERKEHEQMQRDMATLAMQFY
Anja-Hox11/13e  EERDNEQMRRDMANLALKFY
Stpu-Hox11/13d  ERTEQEQIEREKQALGAKFF
Lyva-Hox11/13d  ERTEQEQIEREKQALGAKFY
Pami-Hox11/13d  EKQEQQQIEREKMALGSKYF
Acpl-Hox11/13d  EKQEQQQIEREKMTLGSKYF
Papa-Hox11/13d  ERKEQEEMAREKQSLGAKYL
Apja-Hox11/13d  ERKEQEEMAREKQSLGAKYL
Opsp-Hox11/13d  DKHEAKQMEMEKRALKIDLL
Anja-Hox11/13d  EKQENEQMQREKEALGARML
```

N01

```
Stpu-Hox11/13b  GASSCSLLATFTTTP
Lyva-Hox11/13b  GASSCSLLATFTTTP
Pami-Hox11/13b  GASSCSLLATFTTTP
Acpl-Hox11/13b  GASSCSLLATFTTTP
Papa-Hox11/13b  GTSSCSLLATFTTTP
Apja-Hox11/13b  GTSSCSLLATFTTTP
Opsp-Hox11/13b  GASSCSLLATFTTTP
Anja-Hox11/13b  GPAA-PLL--FTTTP
Sako-Hox11/13b  DVSNCSWLTTFTTTP
Basi-Hox11/13b  DMSNCNWLSSFTTTP
Ptfl-Hox11/13b  DMSNCNWLSTFTTTP
Sako-Hox11/13c  GPSGCGWLTTFTTTP
Basi-Hox11/13c  GPPSCGWLTTFTTTP
Ptfl-Hox11/13c  GPPSCGWLTTFTTTP
```

N02

```
Stpu-Hox9/10  MTTSAFCVNSLINGGDNVTA
Lyva-Hox9/10  MTTSAFCVNSLINGGDNVTA
Pami-Hox9/10  MTTSAFCVNSLINSGESGAI
Acpl-Hox9/10  MTTSAFCVNSLINSGESGAI
Papa-Hox9/10  MTSQGFCVNSLINPVENGGA
Apja-Hox9/10  MTSQGFCVNSLINPVENGGA
Opsp-Hox9/10  MTTTTFCVNSLINSNDAAAI
Mero-Hox9/10  MTASAFCVNSLIHSGENAPV
Sako-Hox9/10  TAGSAFCVNSLISSEENEAL
Basi-Hox9/10  TTTSAFCVNSLISSEESGGI
Ptfl-Hox9/10  TTTSAFCVNSLISSEESGGL
```

N03

**Stpu-Hox11/13d**	**LAHRYSTFYNNLD**
Lyva-Hox11/13d	LTHRYTSFYNNLD
**Pami-Hox11/13d**	**LTCRYPYYPNNLD**
Acpl-Hox11/13d	LTCRYPYYSNNLD
**Papa-Hox11/13d**	**VACRYPYYNNNLD**
Apja-Hox11/13d	VACRYPYYNNNLD
Opsp-Hox11/13d	IPYRYPCY-SNLD
**Sako-Hox11/13b**	**FPCRYPQYYNNMD**
**Basi-Hox11/13b**	**YPCRYPQYYNNMD**
Ptfl-Hox11/13b	YPCRYPQYYNNMD
**Sako-Hox11/13c**	**FHCRYPYY-NNID**
**Basi-Hox11/13c**	**FHCRYPYY-NNLD**
Ptfl-Hox11/13c	FHCRYPYY-NNLD

N04

**Stpu-Hox11/13d**	**YNDE-YSAWNSFDFTSPTTH**
Lyva-Hox11/13d	YNDE-YSAWNSFDFSSPTTH
**Pami-Hox11/13d**	**YGDD-FSVWNSFDFSSATGS**
Acpl-Hox11/13d	YGDD-FSVWNSFDFSSATGS
**Papa-Hox11/13d**	**YHED-FSAWNSFDLATHQQR**
Apja-Hox11/13d	YHED-FSAWNSFDLATHQQR
Opsp-Hox11/13d	YVDDPFSAWNSVESAAFNTQ
Stpu-Hox11/13b	spaaaaaAWNSSEirggaaa
Lyva-Hox11/13b	spaaaaaAWNSSEirggaaa
Pami-Hox11/13b	lspvgggAWTGPDrqfthhh
Acpl-Hox11/13b	lspvgggAWTAPDrqftahh
Papa-Hox11/13b	pisytsNAWNGNDhrQSmay
Apja-Hox11/13b	pisytsNAWNGNDhrQSmay
**Sako-Hox11/13b**	**YNDT-LPSWNSYDFNQSRQC**
**Basi-Hox11/13b**	**YSDS-VPTWNSLDLNQTRQC**
Ptfl-Hox11/13b	YSEA-MPTWNSLDLNQTRQC
**Sako-Hox11/13c**	**HNED-MSTWTTLDFSQSRQF**
**Basi-Hox11/13c**	**HNDE-MSTWTPLDFAQSRQF**
Ptfl-Hox11/13c	HSEE-MSTWTPLDFAQSRQF

N05

**Stpu-Hox9/10**	**GDRPSWLSATS**
Lyva-Hox9/10	GDTPNWLSATS
**Pami-Hox9/10**	**DGTPSWLSATS**
Acpl-Hox9/10	DSTPNWLSATS
**Papa-Hox9/10**	**NEAPSWLSATS**
Apja-Hox9/10	NEAPSWLSATS
**Opsp-Hox9/10**	**GEPPTWLSATS**
**Mero-Hox9/10**	**NETPNWLSATS**
**Sako-Hox9/10**	**GDQPTWLTTTA**
**Basi-Hox9/10**	**NDTPNWMTSAS**
Ptfl-Hox9/10	GDPPNWMTSAS
Stpu-Hox11/13a	SASYTWMAPPS
Lyva-Hox11/13a	SASYTWMAPPP
**Pami-Hox11/13a**	**DTACRWMTTVE**
Acpl-Hox11/13a	DAACRWMTAVD
Papa-Hox11/13a	GGSYSWMSPSQ
Apja-Hox11/13a	GGSYSWMSPSQ
Opsp-Hox11/13a	SSGYPWISSAA
Anja-Hox11/13a	ASGYNWMSPST
Sako-Hox11/13a	SSSYVWIggqq
Basi-Hox11/13a	SSNYVWIggqq
Ptfl-Hox11/13a	SSSYVWIggqq
**Stpu-Hox11/13c**	**HHTPSWMFALA**
Lyva-Hox11/13c	HHTPSWMFALA
**Pami-Hox11/13c**	**GPPPGWMFTLA**
Acpl-Hox11/13c	GPPPGWMFTLA
**Papa-Hox11/13c**	**PHAPGWMFTLA**
Apja-Hox11/13c	PHAPGWMFTLA
**Opsp-Hox11/13c**	**HPPPSWMFALA**
**Mero-Hox11/13c**	**GGPPGWMFTFA**
Stpu-Hox11/13d	SHGSNWVPPSS
Lyva-Hox11/13d	SHGSNWVPPSS
Pami-Hox11/13d	NHLSAWVSSVT
Acpl-Hox11/13d	NHASAWVSSVT
Papa-Hox11/13d	QTTSPWLSTSS
Apja-Hox11/13d	QTTSPWLSTSS
**Opsp-Hox11/13d**	**VHSPGWMSSTV**
Anja-Hox11/13d	PPSGSWVSSLT

N06

**Stpu-Hox11/13b**	**MQIGMEQA-WQAQRP**
Lyva-Hox11/13b	MQIGMEQA-WPAQRP
**Pami-Hox11/13b**	**MQIGMEQG-WSPARP**
Acpl-Hox11/13b	MQIGMEQG-WSPARP
**Papa-Hox11/13b**	**MQIGSEQTNWQAQRT**
Apja-Hox11/13b	MQIGSEQTNWQAQRT
**Opsp-Hox11/13b**	**MQLGMEQS-WSPARP**
Basi-Hox11/13b	MQLQSSTE-Wcggmk
Ptfl-Hox11/13b	MQLQGSSE-Wcggmk
Sako-Hox11/13c	MQWSGAKD-WAVSAA
Basi-Hox11/13c	MQWGSAKE-WSVTAA
Ptfl-Hox11/13c	MQWGSGKD-WSVTAA
Stpu-Hox11/13d	MQNYSPET-WTPVNP
Lyva-Hox11/13d	MQNYSPET-WIPVNP
Pami-Hox11/13d	MQW-NADT—WSATTS
Acpl-Hox11/13d	MQW-NADT-WSATTT
Papa-Hox11/13d	MQW-TPET—WSSPSL
Opsp-Hox11/13d	QQW-NMDS—WCPTTS

N07

Stpu-Hox11/13c RPDCRFLQ
Lyva-Hox11/13c RPDCRFLQ
Pami-Hox11/13c RADCRFVQ
Acpl-Hox11/13c RADCRFVQ
Papa-Hox11/13c RPDCRFIQ
Apja-Hox11/13c RPDCRFIQ
Mero-Hox11/13c RTDCRFVQ

N08

Stpu-Hox11/13b MDSCAAEYRG
Lyva-Hox11/13b MDSCAAEYRG
Pami-Hox11/13b TDSCAAEYRG
Acpl-Hox11/13b TESCAAEYRG
Papa-Hox11/13b TESCASEYRG
Apja-Hox11/13b TESCASEYRG
Opsp-Hox11/13b MESCAAEYRG

N09

Stpu-Hox9/10 MYHLQAEQNASYASPW
Lyva-Hox9/10 MYHLQTDQNTSYASPW
Pami-Hox9/10 MYSLATDQ-SCYTNPW
Acpl-Hox9/10 MYSLATDQ-SCYTNPW
Papa-Hox9/10 MYPIPTDQ-TSYTNHW
Apja-Hox9/10 MYPIPTDQ-TSYTNHW
Opsp-Hox9/10 TLSSAGEQ-TGYPNPW
Mero-Hox9/10 MYSFPTDQ-PGYANPW
Sako-Hox9/10 LYPSGGDQ-NTYTNAW
Basi-Hox9/10 MYPSTGEQNTYTTNAW
Ptfl-Hox9/10 MYPTTGEQNTYTTNAW

N10

Stpu-Hox9/10 GFYS-IGRHQAY-DRY
Lyva-Hox9/10 GFYS-IGRHQAY-DRY
Pami-Hox9/10 SYYT-LNR-QAY-ERY
Acpl-Hox9/10 SYYT-LNR-QAY-ERY
Opsp-Hox9/10 GYYSFGSR-QTYADRY
Mero-Hox9/10 SYYS-LSK-QSY-DRY
Sako-Hox9/10 FYPF-QAR-QGY-DRY
Basi-Hox9/10 FYPF-SAR-QSY-DRY
Ptfl-Hox9/10 FYPF-SAR-QSY-DRY

N11

Stpu-Hox11/13a YVGLMSRFSYH
Lyva-Hox11/13a YVGLMSRFSYH
Papa-Hox11/13a MHGFMGGFPYQ
Apja-Hox11/13a MHGFMGGFPYQ
Sako-Hox11/13a YVGFMSGFPYH
Basi-Hox11/13a YVGFMSGFPYH
Ptfl-Hox11/13a YVGFMSGFPYH

N15/N14 (Examples of N14 are shown as uppercase sequence in echinoderm Hox11/13b)

Stpu-Hox11/13b HQQQYMSSNAYgspyglh
Lyva-Hox11/13b HQQQYMGSNAYgspyglh
Pami-Hox11/13b HQQQYLTSNGFgssygfh
Acpl-Hox11/13b HQQQYLTSNGFgssysfh
Papa-Hox11/13b HQQQYFASGGCsagymns
Apja-Hox11/13b HQQQYFTSGGCsagymns
Opsp-Hox11/13b HQQ-YLP-NGYssaynfh
Stpu-Hox11/13c HQQ-HLSYFNYPSYPTSA
Pami-Hox11/13c TQQ-HLSCLNYPSCANPA
Acpl-Hox11/13c TQQ-HLSCLNYPSCANPT
Papa-Hox11/13c YAP-HLSYINYPSYANPA
Apja-Hox11/13c YAP-HLSYINYPSYANPA
Mero-Hox11/13c GQH-HLPYLNYPSCATSA
Sako-Hox11/13c AQQQYMSSTGYqlqhnpp
Basi-Hox11/13c AQQQYMSSTGYqlqhnpg
Ptfl-Hox11/13c AQQQYMSSTGYqlqhnpg

N17

Pami-Hox9/10 VGSQKGYD
Acpl-Hox9/10 VSSQKGYD
Papa-Hox9/10 MTPQKAYG
Apja-Hox9/10 MTPQKAYG
Opsp-Hox9/10 VSAQKSYS
Mero-Hox9/10 MGTQKNYD
Sako-Hox9/10 IGAQKNYD
Basi-Hox9/10 IGSQKNYD
Ptfl-Hox9/10 IGSQKNYD

N18

Stpu-Hox11/13b GLHNSPYPLDM
Lyva-Hox11/13b GLHNSPYPLDM
Pami-Hox11/13b GFHNSPYPLDM
Acpl-Hox11/13b SFHNSPYPLDM
Papa-Hox11/13b GYMNSPFPLDM
Apja-Hox11/13b GYMNSPFPLDM
Opsp-Hox11/13b NFHNSAYPLHD
Sako-Hox11/13c LQHNPPYPMNM
Basi-Hox11/13c LQHNPGYPMNM

2. Example sequences showing the typical locations of conserved motifs within the peptide. Homeodomains are bolded. Predicted or known exon boundaries are underlined. Motifs are highlighted in grey (light grey for divergent or partial matches); motif IDs are written above each motif.

a. Pami-Hox9/10

N02
MTTSAFCVNSLINSGESGAILKSGQQTDIHGSPGLAATKCASGSGSFAPKFADSPAQAVAGHNMPP
N09 N17
AAGPPSATNMYSLATDQSCYTNPWLYSSGDMTHYPPMSVAGVSEDYENNPSYPYVGSQKGYDTALV
N10
PSSGSYYTLNRQAYERYTNHSTNVYHTSAHNGHGLPASFAGSFAAKFVSGTADTDRQRYGNFDSYD
N05
SPTFGTSVKAGQQGSGKTQTAYTAAPATGNTSPPLGKESSKQTTTSTESVTKSEDDDTTKTDGTPS

WLSATS**GRKKRCPYTKYQTLELEKEFLFNMYLTRDRRVDIARMLNLTERQVKIWFQNRRMKMKKMH**

RAQILSC

b. Stpu-Hox11/13a

N11

MEGLQAPRAFPHNIGTFYDTVANGHNNGYNLSHDGSYVGLMSRFSYHSSTNCLRGYPSPAGGGGGG

GGGGGVDAESSSAGCCSSTAAITPTNCANNGGHSWTSPPPNHGDKPAGHFPFFYQEVYTPGVHHSP

N05

PTSLGHHVLSTHGKHGYSSGSANPHPACGNGDDDPSRPESDSDHSVSHQAVSASYTWMAPPSNVR**T**

RKKRKPYTKFQTFELEKEFLYNMYLTRDRRSHISRALSLTERQVKIWFQNRRMKLKKMRAREENER

KNHSHHPAGQHHPQHHHHLESKGGDHCSIGGGGATELIHKPSAIHHAYVGGGGGGGGTHESNPLAE

LQQHGVLQHHGVSSI

c. Stpu-Hox11/13b

N06 N08

MQIGMEQAWQAQRPTGMDSCAAEYRGISAPMNGLYGTSSRQRASCNAMVSVGSSNPEQLSAFHYSY

N04

PMYNSPTESSLAASSAGDELPPGAAGSGVSPAAAAAAWNSSEIRGGAAAAAAAAAHGATPRPYANT

N14 N18 N01

FGNTFLTHAGSPPHHAHMAHQQQYMSSNAYGSPYGLHNSPYPLDMTGGASSCSLLATFTTTP**RRTK**

RRPYSKLQIYELEKEFTTNMYLTRDRRSKLSQALDLTERQVKIWFQNRRMKMKKLNDKEKTQSTKK

KSAEGSSHTSSTTSSTSSTSSTSSASTMSQSSSSQHQQQQQHHHHQHNHHHHNHHPLPVAINTSHP

SVGHQPQLR

d. Basi-Hox11/13b

N06 N03

MLATENAPFLNTNKMQLQSSTEWCGGMKTSMDRLSALFREKQNATDNGYANMSSEQNIYPCRYPQY

N04

YNNMDPANGTYSDSVPTWNSLDLNQTRQCLNGFQPSSMYSGGGSIAQHHPYANTNYGVHPSAYTGN

N01

VPGVTDMSNCNWLSSFTTTP**RRTKRRPYSKMQIYELEKAFQQNAYLTRERRQKYSQQLNLTERQVK**

IWFQNRRMKSKKQTEREKMEEKERREHEQSMDMVHSS

e. Pami-Hox11/13c

MFYDLFCNSSTRTTPSSDLAMENGPQTFGGKPCALASPSCSVSYTTARAATGTGFMQVPAPAVERG

N07

PSTPTSHLHHPSAPGPGGFAYVNTRADCRFVQPGAASASSSYDEQYKSYRSASALSFNSMELSTGH

N15

EAGHHRMDFGGPMRYSGSMGGATAAAVMYGAASNHQATQQHLSCLNYPSCANPAGYPFGISGTDGP

N05

PPGWMFTLAALP**RRTKRRPYSKMTIFELEKEFQAHQYLTRDRRARLAQSLSLTERQVKIWFQNRRM**

KQKKVNDKEKTKAAGKTDRKSD

f. Basi-Hox11/13c

N06 N03

MQWGSAKEWSVTAANTDTLAQCFTARPSAMTSMFNRSDNGGLMSTQSGSDQHASFHCRYPYYNNLD

N04 N14 N18

AAAAAAATGGHNDEMSTWTPLDFAQSRQFANSFNHHHHPAMLNTSPTAAQQQYMSSTGYQLQHNPG

N01

YPMNMTGPPSCGWLTTFTTTP**RRTKRRPYSKLQIFELEKEFQQNMYLTRDRRTRLSQTLNLTERQV**

KIWFQNRRMKLKKMTEREHSEQEVLRQQQQQPQQPQQQSQTQQQSTQQQQQQQQQQTTQTVVA

g. Pami-Hox11/13d

N06 N03

MQWNADTWSATTSVETGTTSQLFASRTPSLSSVYNKCSVVGESGAGPGGATHQPVPLTCRYPYYPN

N04

NLDPGSSSSALASISPYGDDFSVWNSFDFSSATGSQRYHSPFASPAAPTPLNPAAAAAAAAVSSHQ

N05

HTRDSYLSGASGAGGYSSVSHRSTNYPLSVSGSSNHLSAWVSSVTSSP**RRTKRRPYSKLQIIELEK**

C01

EFQDNMYLTRDRRTRLAEVLNLTERQVKIWYQNRRMKMKKMTEREKQEQQQIEREKMALGSKYFAT

HHH

h. Pami-Hox11/13e

MQQWPANRPRCLGVTDAAVEPALLLNYGEYDSFERSPTVWEDAPPVTGSCMYTSTISRAVVPPSCQ

HQNYYDTLAAPAASYVSSASSIQLKYPAHTATVSSTFTDALLADDQQDSEVLQSPDAVSYHTSPLM

DDGSHVEHSVSTSLKPGG**SRPKRRPYSKLQLLHLEQEFQRSMYPCRERRAWLSQVLSLTERQVKIW**

C01

FQNRRTKLKRTTEREQRENAQMQRDMANLAMRFYDPAQ
